# Evaluation of thyroid dysfunction in pregnant women with gestational and pre-gestational diabetes

**DOI:** 10.12669/pjms.292.2862

**Published:** 2013-04

**Authors:** Hajieh Shahbazian, Nahid Shahbazian, Mahnaz Rahimi Baniani, Leila Yazdanpanah, Seyed Mahmuod Latifi

**Affiliations:** 1 Hajieh Shahbazian, Endocrinologist, Associate Professor, Diabetes Research Center, Ahvaz Jundishapur University of Medical Sciences, Ahvaz, Iran.; 2 Nahid Shahbazian, Gynecologist, Associate Professor, Diabetes Research Center, Ahvaz Jundishapur University of Medical Sciences, Ahvaz, Iran.; 3 Mahnaz Rahimi Baniani, MD, Diabetes Research Center, Ahvaz Jundishapur University of Medical Sciences, Ahvaz, Iran.; 4 Leila Yazdanpanah, MD, Diabetes Research Center, Ahvaz Jundishapur University of Medical Sciences, Ahvaz, Iran.; 5 Seyed Mahmuod Latifi, MSc in Biostatistics, Diabetes Research Center, Ahvaz Jundishapur University of Medical Sciences, Ahvaz, Iran.

**Keywords:** Thyroid dysfunction, GDM, Pregestational DM, Anti TPO Ab, Anti TgAb

## Abstract

***Objective: ***The aim of this study was to investigate thyroid function tests in Gestational Diabetes Mellitus (GDM) and pre-gestational DM and control group.

***Methodology*** *: *There were 61 pregnant diabetic women in study group and 35 pregnant women in control group. Serum T4, T3, T3RU, FTI, TSH and Anti TPO Ab were assessed in each person.

***Results*** *: *About 36% of patients had GDM and 64% pre-gestational DM. Thyroid dysfunction was detected in 18% of study group compared with 8.6% of control group (P = 0.2). There was Thyroid dysfunction in 4.5% of GDM and 25.6% of pregestational DM (P = 0.045). There was no statistically significant difference between thyroid dysfunction in GDM group and control group (P=0.99).27% of GDM and 36% of pregestational DM and 23% of control group had positive titer of Anti TPO Ab without statistically significant differences among the three groups.

***Conclusion*** *:* Thyroid dysfunction is prevalent in women with pre-gestational DM so, thyroid function should be evaluated in these patients during pregnancy. Rate of thyroid dysfunction in GDM patients is similar to normal pregnant control women. High prevalence of positive titer of TPO Ab was seen in diabetic and non-diabetic pregnant women.

## INTRODUCTION

Diabetes is one of the most prevalent endocrine disorders during pregnancy. It is classified as gestational diabetes (diabetes which was first diagnosed during pregnancy) or pre-gestational diabetes. GDM occurs in 2-6% of pregnant women in North America that is estimated 10 times more than pre-gestational diabetes.^1^^,^^2^

In Iran, the prevalence of gestational diabetes has been reported between 4.7-7.4% in different regions that according to the new ADA criteria, the prevalence of gestational diabetes will be much higher than the above numbers.^3^^-^^5^About 10-15% of pregnant women have thyroid dysfunction during the first half of pregnancy, which may be hypothyroidism or hyperthyroidism.^6^ However, the prevalence of thyroid dysfunction in pregnant women with type I diabetes is about three times more than general population and subclinical hypothyroidism is more prevalent.^7^

In some studies 40% of pregnant women with type I diabetes may also have thyroid dysfunction simultaneously.^8^ Some studies have reported high incidence of hypothyroxinemia and high Anti TPO titer in GDM.^9^ Prevalence of high titer of Anti TPO Ab in healthy pregnant women and type 1 diabetes also have significant difference.^10^ Delayed diagnosis of hypothyroidism in pregnancy leads to congenital malformations and respiratory distress in newborns of these mothers.^11^

Furthermore mother's diabetes during pregnancy can influence the secretion of T3 or conversion of T4 to active T3 in the fetus and premature newborns.^12^ When anti-thyroid antibodies are positive, there is risk of premature rupture of amniotic sac and preterm labor.^13^ Some studies have showed that even without the overt thyroid dysfunction, autoimmune thyroid disease increases abortion rates up to 3 to 5 times.^14^

The purpose of this study was to evaluate thyroid function tests and thyroid autoantibody level in pregnant patients with diabetes (GDM and pre-gestational diabetes) and comparison with a non-diabetic control group in Ahvaz (Western South of Iran).

## METHODOLOGY

This is a cross sectional prospective study. The study population was pregnant women with diabetes (pre-gestational diabetes and gestational diabetes) who were referred to Ahvaz Golestan Hospital Endocrinology Clinic from 2004 to 2006. Diabetic patients were aged 19 to 47 years. Thirty five non-diabetic pregnant women were also considered as control group. Sequential sampling was conducted. Written consent was obtained from all patients and the study was approved by the Ethics Committee of Ahvaz Jundishapur University of Medical Science.

In this study T3, T4, TSH, T3RU and Anti TPO Ab were measured in all GDM and pre-gestational diabetic pregnant women and control group. T3, T4, T3rU, FTI hormones was measured by RIA method, TSH by IRMA, TPO Ab by ELISA, Counter. Kits used in this laboratory was Immunotech product of A Beckman coulter company made in America.

Pre-gestational diabetes had been distinguished before pregnancy. GDM in pregnant women had been considered if two of the four criteria were detected following 100 g glucose tolerance test:(FBS ≥ 95, 1h BS ≥ 180,2hBS ≥ 155, 3hBS ≥ 140 mg/dl).

In the present study individuals with normal levels of T4 and T3 and TSH <0.3mlU / L had been considered as sub clinical hyperthyroidism and individuals with normal T4 and T3 and TSH> 4mlU / L as sub clinical hypothyroidism. High T4 and TSH <0.3 as hyperthyroidism and T4 <4.8 and TSH>4 as hypothyroidism.

Questionnaire included type of diabetes (GDM or pre-gestational), thyroid disease history, history of drug consumption for thyroid dysfunction, gestational age at diagnosis was completed for each patient. The data obtained in this study were analyzed by statistical software SPSS15.

## RESULTS

This study was performed on 61 diabetic pregnant patients. Among them 22 patients (36%) had GDM and 39 (64%) had pre-gestational diabetes (20% type 1 and 80% type 2 diabetes). Control group were 35 non-diabetic pregnant women. Diabetic patients were aged 19 to 47 years. The overall mean age was 30±6.5 years in all diabetic patients and 28.9 ± 5.53 years in GDM and 30.6±7.10 years in pre-gestational diabetes. Mean age of control group was 27.7±4.25. 

Among 22 patients with GDM, 21 patients (95.5%) had normal thyroid function and one patient (4.5%) had subclinical hypothyroidism. Among 39 patients with pre-gestational diabetes, 29 cases (74.4%) had normal thyroid function and 10 cases (25.6%) had thyroid dysfunction. In control group among 35 patients, three patients (8.6%) had thyroid dysfunction.

Overall Subclinical hypothyroidism was the more prevalent thyroid dysfunction in the three groups. Thyroid dysfunction was significantly higher in pre-gestational than GDM group (P = 0.037). Thyroid dysfunction in GDM and pre-gestational group did not have significant difference with control group (p =0.99, 0.054 respectively). Previous history of thyroid disease were found in 16 cases (16.3%) and all of them were in pre-gestational group.

Types of thyroid dysfunction in diabetic patients and control groups are shown in Table-I.

High titer of TPO Ab was detected in 27% of the GDM group, 36% of pre-gestational DM and 23% of control group. There was no significant differences in Anti TPO positive titer between three groups (p=0.188).

**Table-I:** Thyroid dysfunction in GDM, pre gestational DM and control group

*Thyroid function* *Groups*	*No. of Normal *Thyroid function (%)*	*No. of Hypothyroid* *(%)*	*No. of Subclinical* *Hypothyroid* *(%)*	*No. of hyperthyroid* *(%)*	*No. of Subclinical* *hyperthyroid* *(%)*
GDM	21(95.5%)	0(0%)	1(4.5%)	0(0%)	0(0%)
Pre-gestational DM	29(74.4%)	1(2.6%)	4(10.3%)	1(2.6%)	4(10.3%)
Control group	32(91.4%)	1(2.86%)	1(2.86%)	1(2.86%)	0(0%)

*No=number

## DISCUSSION

About 10-15% of pregnant women have thyroid dysfunction during the first half of pregnancy.^6^ Patterns of ontogeny of fetal cerebral cortex deiodinases and thyroid hormone receptors that begin by 7-8 weeks' gestation, can be an evidence that thyroid hormone is important in fetal neurodevelopment.^15^

Thyroid dysfunction specially mild or subclinical hypothyroidism is associated with impaired neurodevelopment in the offspring and there is a link between this problem and mental retardation in the newborn.^16^^-^^18^

Sixty one diabetic pregnant women were enrolled in this study, 22 patients (36%) were diagnosed as GDM and 39 patients (64%) had pre-gestational diabetes. There were 35 healthy pregnant women as control group. Results showed that 25.6% of pregnant women with pre-gestational diabetes and 4.5% of women with GDM and 8.6% of control group had thyroid dysfunction. That sub clinical hypothyroidism was more prevalent. Thyroid dysfunction in GDM patients had no significant difference with control group. In Luisa Ruas et al study the prevalence of thyroid dysfunction in GDM was 5.1% that is comparable with our study.^19^

A study in normal pregnant women in Iran reported thyroid dysfunction prevalence as 2.6% that is similar to our study.^20^ Agarwal et al study reported that thyroid dysfunction rate is similar in GDM and non-diabetic pregnant women,^9^ but Velkoska Nakova et al reported higher prevalence of hypothyroxinemia in GDM than non-diabetic controls.^10^

In Gllas PR and Lois Jovanic P studies thyroid dysfunction in pregnant women with type 1 diabetes was 40%^7^ and 40.9%^8^ respectively; that is higher than our study. The cause of this difference may be because most of our patients had type 2 diabetes (80%) rather than type 1 diabetes (20%), whereas in Gllas PR and Lois Jovanic P study all patients had type 1 diabetes and thyroid dysfunction is higher in this group.^7^^,^^8^ In another study in non-pregnant diabetic women in Iran the prevalence of thyroid dysfunction was 40% in type 1 and about 20% in type 2 DM.^21^^,^^22^

Gonzalez et al. also showed that 26% of pregnant women with type 1 diabetes and 4% of healthy pregnant women had thyroid dysfunction which is comparable with our study.^23^ However, Di Gilio AR and colleagues study showed no significant difference between TSH, FT4, FT3 levels in diabetic and healthy pregnant women.^24^ In this study sample size was 15 women with type 1 diabetes and 13 healthy persons, which was small and may affect the results. Gllas PR also showed the same frequency of clinical hypothyroidism as our study.^7^

Antithyroid antibodies can cross the placenta and therefore they can influence fetal brain and neurodevelopment.^16^^,^^17^ Some studies showed that even without the overt thyroid dysfunction, autoimmune thyroid disease increases abortion rates up to 3 to 5 times.^14^

 In the present study, positive TPO Ab was seen in 27% of women with GDM and in 36% of pregnant women with pre-gestational diabetes and 23% of control group and there was no statistically significant difference between them.

Ortega-Gonzalez et al studied 50 healthy pregnant women, 50 GDM women and 50 pregnant women with type 2 diabetes TPO Ab> 251U/ml ( strongly positive) was found in 10% of healthy women, 10% of pregnant women with type 2 diabetes and 6% in women with GDM without statistically significant difference.^25^ Luisa R et al study evaluated Tg Ab and Anti TPO Ab in 408 GDM women and reported high titer of antibodies in 5% of study population.^19^ The difference between mentioned studies and our study could be due to regional differences, race and iodine consumption. Because of consumption of iodine salt for correction of iodine deficiency in our country, it can increase thyroid auto-immunity and rise in thyroid auto antibodies titer.^14^ In Ortega study just TPO Ab > 251 U / ml (strongly positive) was considered, whereas in our study all patients with positive TPO Ab, have been considered. Bech et al also reported 17.85% positive titer of anti-microsomal antibody in a group of type 1 diabetes pregnant women.^26^

Positive titer of auto antibody in pregnancy may be predictive of higher prevalence of post-partum thyroiditis in our population. Therefore for long term prospective studies to follow up of patients with positive antibodies and subclinical Thyroid dysfunction after delivery is recommended.

## CONCLUSION

This study showed that thyroid dysfunction has a high prevalence in women with pre-gestational DM and therefore thyroid function should be evaluated in these patients during pregnancy. Thyroid dysfunction was equally prevalent in women with GDM and normal pregnant women but 27% of them had positive titer of TPO Ab that warrants follow up for post-partum thyroiditis, dysfunction.
